# Domoic Acid Risk and the Potential of Meta‐Omics for Environmental Surveillance

**DOI:** 10.1111/gcb.70593

**Published:** 2025-11-04

**Authors:** Enrico Bortoletto, Umberto Rosani

**Affiliations:** ^1^ Department of Biology University of Padova Padova Italy

**Keywords:** domoic acid, metagenomics, meta‐omics, metatranscriptomics, pseudo‐nitzschia

Harmful algal blooms (HABs) represent one of the current ecological and public health challenges, likely to become more severe under the anthropogenic‐mediated climate change scenarios. Domoic acid (DA), a neurotoxin produced by roughly half of the known species of the diatom *Pseudo‐nitzschia*, can bioaccumulate in shellfish species causing amnesic shellfish poisoning (ASP), with potentially lethal consequences for marine birds and mammals, as well as for other shellfish consumers during HABs, like humans (Krasner et al. 2025). The 2015 HAB event, which occurred along the US Pacific coast, is probably the most severe on record, with a wake of economic consequences for the prolonged closures of valuable fisheries (McCabe et al. 2016). Since climate change is reshaping marine ecosystems, the frequency, distribution, and severity of HAB events are projected to increase. Continuous research is therefore essential to provide new insights into *Pseudo‐nitzschia* biology and global distribution as well as into DA production pathways striving for a comprehensive and integrated understanding of algal blooms in the oceans (Litchman 2023).

Considering this context, the recent study published on GCB by Dong Xu et al. (2025) provides a landmark contribution. The authors applied a multi‐omics approach, analyzing 18S metabarcoding, shotgun metagenomic and metatranscriptomic data of water filtrates obtained from the Tara Oceans as well as from targeted sampling along the Chinese coast and around the Antarctic Circle (*SOPICC project*). This integrated strategy enabled the detection of *Pseudo‐nitzschia* species and the identification of active DA biosynthetic and transport pathway genes (i.e., *dabA*, *dabB*, *dabC*, *dabD*, and *slc6*). Their results provided an unprecedented perspective on the global distributions of four highly toxic *Pseudo‐nitzschia* species, with DA‐associated genes found in open‐ocean environments from pole‐to‐pole, with water temperatures ranging from −1.81°C to 31.2°C. This emerges as a novelty, revealing that these diatom species are not only abundant in coastal areas but are rather cosmopolitan in the oceans.

Furthermore, the authors have performed an 800‐day laboratory evolution experiment maintaining *P. multiseries* at different temperatures (ranging from 5°C to 30°C) to measure physiological responses and calculate the DA production rate. They predicted that the toxicity of *Pseudo‐nitzschia* could increase by 200% by the end of the century under the SSP2‐4.5 climate scenario.

These findings carry profound implications for seafood safety, marine wildlife conservation, and ecosystem resilience. Xu et al. results support temperature as a dominant driver of *Pseudo‐nitzschia* distribution and toxicity, while also showing that nutrient ratios and pH, modulate toxin production. This complexity underscores why forecasting harmful blooms remains challenging: not only do species differ in their toxin profiles, but also strains within a species can vary dramatically in their responses to environmental parameters.

Nevertheless, traditional monitoring methods struggle to capture this diversity in real time, making molecular forecasting (i.e., meta‐omics), although expensive, necessary to move forward HAB prediction and modeling (Brunson et al. 2024). Indeed, meta‐omics offers a path forward by providing a detailed picture of the identity and functional potential of biological communities through metabarcoding and metagenomics, respectively, as well as revealing the pool of active (i.e., transcribed) genes via metatranscriptomics. Disentangling the meta‐community, unveiling the potential biosynthetic and metabolic functions and tracing the active molecular pathways will ease the coupling of molecular signals with environmental drivers, with not neglectable advantages for mechanistic modeling. Beyond classical microscopy and pigment analysis, omics science has the potential to indicate “what may happen next.” Xu et al. demonstrate this by identifying biosynthetic genes, such as *dabA–D* and the transporter *slc6*, across global datasets. The detection of DA markers can bridge the gap between species presence and toxin release, offering the prospect of early warning systems that forecast risk before human or ecological impacts are felt (Brunson et al. 2018).

From a broader perspective, the integration of omics within global observing frameworks could transform HAB science as remote sensing transformed studies of phytoplankton biomass decades ago (Blondeau‐Patissier et al. 2014). It is now feasible to embed metagenomic surveys into routine oceanographic cruises, aquaculture monitoring, and coastal observing systems. These surveys can detect cryptic species and strain‐level diversity that would otherwise remain invisible, including microeukaryotes, prokaryotes, and viruses, while also building the reference databases needed for more accurate taxonomic and functional annotations (de Vargas et al. 2015; Duan et al. 2024). The key challenge is no longer whether omics approaches can provide ecological insight—they clearly can—but how to translate these insights into operational monitoring and policy. Brunson et al. (2024) exemplified this transition by linking transcriptomic profiles with DA production to achieve molecular forecasting at coastal scales, whereas Xu et al. (2025) extended this paradigm to global meta‐omics data, providing a climate‐sensitive framework for toxin prediction. Accordingly, a substantial investment in sequencing infrastructure, standardized and ameliorated protocols and bioinformatic pipelines, and open data repositories is required to capture the genetic and functional diversity of toxigenic *Pseudo‐nitzschia* (Fujiyoshi et al. 2023; Brunson et al. 2024).

While Xu et al. represents an outstanding advancement in HAB research, some caveats deserve consideration. Meta‐omics datasets such as those generated by the Tara Oceans and SOPICC projects, though extensive, still provide limited temporal resolution and uneven spatial coverage. Short‐lived bloom dynamics and regional variability in DA biosynthesis may therefore remain underrepresented, particularly in poorly sampled tropical and upwelling systems. Moreover, the presence or even the expression of DA biosynthetic genes does not necessarily equate to active toxin synthesis. Future research could benefit from synergistic multi‐omics analyses, integrating ribosome profiling (Ribo‐seq) with *Pseudo‐nitzschia* genomics or coupling targeted proteomics with metatranscriptomic datasets, to further deepen the diatom's biology, validate the actual translation of toxin‐related genes, and quantify protein‐level activity under changing environmental conditions. Such multi‐omic approaches would provide a stronger mechanistic link between gene expression and metabolic output. Long‐term, time‐series meta‐omics surveys combined with direct DA quantification, multi‐strain physiological experiments, and the integration of omics‐derived predictors into coupled oceanographic models will be crucial for validating and refining predictive frameworks for *Pseudo‐nitzschia* toxicity in a warming ocean.

In sum, Xu et al. (2025) have demonstrated that the risk posed by DA is global and likely will intensify. Their work highlights the power of meta‐omics to reveal hidden ecological patterns and to anticipate future threats under climate change. As HABs expand in frequency and severity, the ability to trace and predict DA production through molecular surveillance will become essential. The next step is to embed these technologies within global monitoring frameworks, shifting from retrospective assessment to proactive management. Protecting marine ecosystems and human health in the 21st century will increasingly depend on our willingness to embrace omics‐based monitoring, as a cornerstone of global change biology.

## Author Contributions

**Enrico Bortoletto:** writing – original draft, writing – review and editing. **Umberto Rosani:** writing – original draft, writing – review and editing.

## Conflicts of Interest

The authors declare no conflicts of interest.

## Linked Articles

This article is a Invited Commentary on Xu et al., https://doi.org/10.1111/gcb.70384.

## Data Availability

Data sharing not applicable to this article as no datasets were generated or analysed for the current article.
